# Supplementary material for: Changes in Faecal Microbiota Profile and Plasma Biomarkers following the Administration of an Antioxidant Oleuropein-Rich Leaf Extract in a Rat Model Mimicking Colorectal Cancer
Source: Antioxidants (Basel). 2024 Jun 14;13(6):724. doi: 10.3390/antiox13060724 (PMC11200411; doi:10.3390/antiox13060724)
Supplement: Supplementary file 1 [file antioxidants-13-00724-s001.zip › Supplementary figures.pdf]

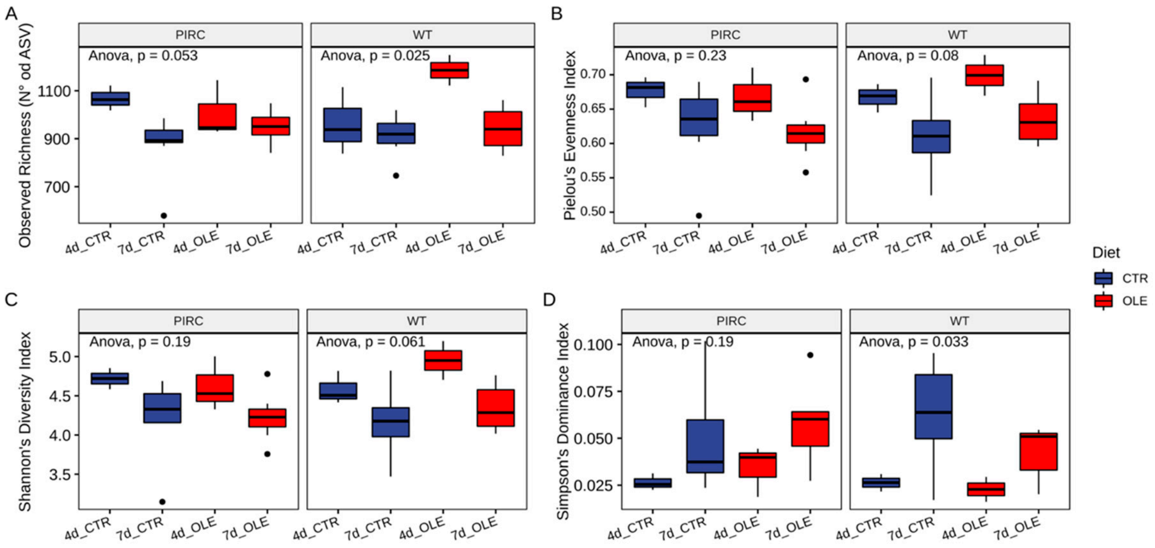

Figure S1. Alpha diversity analysis of bacterial communities in rats, calculated by number of observed ASVs, Pielou's Evenness, Shannon and Simpson indexes. (A) Differences in observed Richness between samples groups at 4 days and 7 days, with and without ORLE in PIRC and WT genotypes assessed with t-test. (B) Differences in Pielou's Evenness diversity index between sample groups at 4 days and 7 days, with and without ORLE in PIRC and WT genotypes assessed with t-test. (C) Differences in Shannon's diversity index between sample groups at 4 days and 7 days, with and without ORLE in PIRC and WT genotypes assessed with t-test. (D) Differences in Simpson's dominance index between samples groups at 4 days and 7 days, with and without ORLE, in PIRC and WT genotypes assessed with t-test.

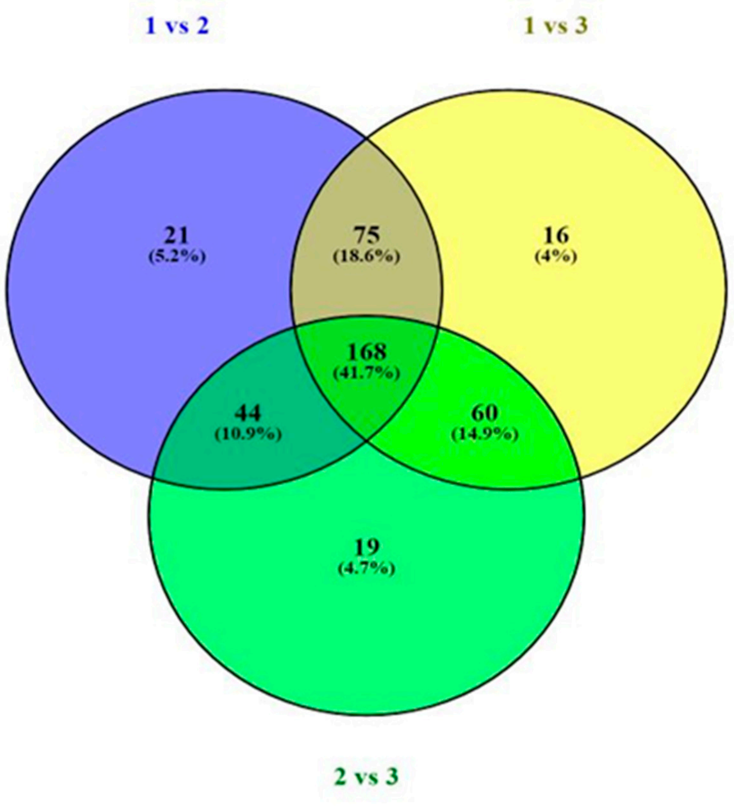

Figure S2. Venn diagram showing the number of common and exclusive VIP discriminant metabolites related to the different comparisons, namely “1 vs 2” (PIRC-ORLE vs PIRC-CTR), “1 vs 3” (PIRC-ORLE vs WT-CTR), and “2 vs 3” (PIRC-CTR vs WT-CTR).
